# A Prediction Method for Animal-Derived Drug Resistance Trend Using a Grey-BP Neural Network Combination Model

**DOI:** 10.3390/antibiotics10060692

**Published:** 2021-06-09

**Authors:** Xinxing Li, Ziyi Zhang, Ding Xu, Congming Wu, Jianping Li, Yongjun Zheng

**Affiliations:** 1 Beijing Advanced Innovation Center for Food Nutrition and Human Health, College of Information and Electrical Engineering, China Agricultural University, Beijing 100083, China; lxxcau@cau.edu.cn (X.L.); S20203081452@cau.edu.cn (Z.Z.); 2 Beijing Advanced Innovation Center for Food Nutrition and Human Health, College of Engineering, China Agricultural University, Beijing 100083, China; 386048263@alu.cau.edu.cn (D.X.); ljping@cau.edu.cn (J.L.); 3 College of Veterinary Medicine, China Agricultural University, Beijing 100083, China; wucm@cau.edu.cn

**Keywords:** drug resistance, microbial, BP neural network, grey system, GM(1,1)-BP neural network model

## Abstract

There is an increasing drug resistance of animal-derived pathogens, seriously posing a huge threat to the health of animals and humans. Traditional drug resistance testing methods are expensive, have low efficiency, and are time-consuming, making it difficult to evaluate overall drug resistance. To develop a better approach to detect drug resistance, a small sample of Escherichia coli resistance data from 2003 to 2014 in Chengdu, Sichuan Province was used, and multiple regression interpolation was applied to impute missing data based on the time series. Next, cluster analysis was used to classify anti-*E. coli* drugs. According to the classification results, a GM(1,1)-BP model was selected to analyze the changes in the drug resistance of *E. coli*, and a drug resistance prediction system was constructed based on the GM(1,1)-BP Neural Network model. The GM(1,1)-BP Neural Network model showed a good prediction effect using a small sample of drug resistance data, with a determination coefficient R^2^ of 0.7830 and an RMSE of only 0.0527. This model can be applied for the prediction of drug resistance trends of other animal-derived pathogenic bacteria, and provides the scientific and technical means for the effective assessment of bacterial resistance.

## 1. Introduction

With continued economic development, China’s output of animal-derived food has been increasing. In 2018 alone, China’s output of animal-derived food reached 214 million tons. However, with the rapid increase in production, the problem of drug resistance has worsened. The incidence of *Escherichia coli* (*E. coli*) disease in chicken farms has reached 30–70%, with mortality even as high as 60%. The pig industry is no better, with the highest incidence of *E. coli* in pigs [1,2]. Numerous studies have shown that the use of animal antibiotics accounts for more than half of the total consumption, and that resistance to antibiotics of animal origin can be transmitted to humans directly or indirectly through the food chain [3]. Drug resistance seriously affects the effective prevention and treatment of infectious diseases and poses a significant threat to the health of animals and humans [4,5,6,7].

Some regions and countries have established specialized databases tracking drug resistance at the national, laboratory, farm, and other levels, providing a large amount of valuable data and analysis of drug resistance [8,9,10,11,12,13]. The first drug resistance surveillance system was established in China only in 2009 [3,14]. Additionally, the mining of drug resistance data is still not deep, lacking deep correlation analysis, which makes it difficult to predict the development trend of drug resistance under multiple factors [15].

Several scholars have explored machine learning algorithms for drug resistance [16], including identifying factors associated with antibiotic misuse [17] and detecting gene sequences that differ from known drug resistance genes [18]. Machine learning can automatically classify a large and irregular collection of samples into different categories, add labels to different categories based on the commonalities of the data, and then use them to train a classifier. Thus, machine learning algorithms can learn drug resistance mechanisms from data and predict resistance to various antibiotics without any prior information. Liu et al. used support vector machine (SVM) and set covering machine (SCM) models to learn and predict drug resistance for five drugs (tetracycline, ampicillin, sulfisoxazole, trimethoprim, and enrofloxacin), and the training accuracy and testing accuracy of the SVM and SCM models for the five drugs were above 90% [19]. Maguire et al. analyzed AMR in nontyphoidal *Salmonella* isolates from chicken farms and generated logistic regression models to predict the observed drug resistance phenotype with an accuracy of 92–99% [20]. Kulshrestha et al. constructed decision tree classifiers based on machine learning and data mining techniques to identify resistance patterns based on results from patients who underwent antimicrobial susceptibility testing and used them to predict resistance to various antibiotics [21]. Elshayeb et al. used logistic regression linear equations to predict the epidemic potential of multi-drug resistant *Salmonella typhimurium* in Sudan [22]. Bhatnagar et al. established a seasonal, autoregressive integrated moving average model to predict the monthly incidence of dengue and hemorrhagic fever [23]. Lu et al. developed a Markov chain model to predict the outbreak of infectious diseases [24].

Moreover, Van Boeckel et al. addressed the problem of quantitatively measuring global livestock antibiotic consumption by mapping antibiotics use in food animals in 2010 and 2030 using a Bayesian statistical model that combines livestock density maps, economic projections of meat product demand, and current estimates of antibiotic consumption in high-income countries [25]. Li et al. established a DRI for anti-*E. coli* drug risk status based on principal component analysis, combining AMR, antibiotic use data, and environmental factors (water, soil) to reveal the effects of antibiotic use, contamination level, and drug resistance, bridging the gap between judging drug resistance by resistance rate alone [26]. Maldonado et al. analyzed drug resistance from 2007 to 2012 based on cardinal linear trends and summarized the bacterial species that changed significantly [27].

Machine learning and data mining techniques [28,29,30] can be well suited to analyze diverse and fragmented drug resistance datasets and reveal their mechanistic changes. However, the above statistical prediction methods have limitations, requiring large amounts of data according to specific statistical methods, and are not suitable for modeling and analysis based on small sample datasets. 

The following prediction models are developed for small sample data and incomplete data. Fan et al. established a grey model GM(1,1) and used a time series analysis method to fit and predict the trend of ceftazidime resistance in *E. coli* [31]. Shen et al. used discrete grey model DGM(1,1) to predict the incidence of typhoid fever [32]. Shu et al. constructed a combination model of a grey model and a neural network model, fitted the aminoglycoside resistance index of *Klebsiella pneumoniae*, and found that the incorporation of the grey neural network improved the stability and reliability of both the fitting and prediction results relative to those of the single model [33].

In summary, current prediction models of drug resistance based on data analysis have improved prediction results, but these models remain limited [34]. In this paper, we established drug resistance prediction models for different years based on the drug resistance data of small samples of *E. coli* in Sichuan Province-Chengdu City from 2003 to 2014, and achieved good prediction results. Based on these models, we established a drug resistance prediction system for animal-derived pathogenic bacteria to analyze the changes of drug resistance under different years. The drug resistance prediction model established in this study for small sample data can be used to predict drug resistance trends in other animal-derived pathogenic infectious diseases, which is of great significance for protecting the health of animals and humans and curbing the growth and spread of drug resistance.

## 2. Materials and Methods

### 2.1. Data

This section describes the data used in this study, the preprocessing steps of the obtained drug resistance data, and the use of a multiple regression interpolation method to interpolate the missing drug resistance data. Cluster analysis is then used to classify the drugs used against *E. coli*, providing the data basis for the prediction model [35].

#### 2.1.1. Data Sources

In this study, a total of 11,184 strains of *E. coli* were collected from the Chengdu Monitoring Station of Sichuan Province from 2003 to 2014, the drug resistance potential of ofloxacin, enrofloxacin, doxycycline, tetracycline, gentamicin, ceftiofur, and sulfafurazole were assessed, and preliminary processing of these data was performed. The dataset in this paper mainly includes the number of *E. coli* strains that are resistant to each of the seven drugs mentioned above.

#### 2.1.2. Data Preprocessing

Most drug resistance data suffer from insufficient monitoring for a discrete time period or for a specific region. An additional complication is that there may be missing values in multiple regions for many years, which will inevitably increase the difficulty of statistical analysis. To analyze and process drug resistance data and improve the predictive effect of drug resistance, these missing values must first be determined.

Data for 2005 and 2006 were missing from the *E. coli* drug resistance data collected in this study, while a few data for other years were missing. Based on the characteristics of the time series of the drug resistance data, a multiple linear regression interpolation method was used. This method is a single interpolation, which uses the missing variables and related variables in the missing set to establish a regression model to generate a set of estimated values, and then uses the estimated values to fill in the missing set to obtain the complete set. This is performed as follows.

(1) Select variables to establish a multiple linear regression model [36], let *y* represent the missing value of resistance. The data of drug resistance in other years were represented as $x_{1},\;x_{2},\ldots$, and $x_{n}$ represents the variable associated with the missing value. The multiple linear regression model is written as Equation (1):

(1) $$y=\beta_{0}+\beta_{1}x_{1}+\beta_{2}x_{2}+\cdots +\beta_{n}x_{n}+\varepsilon \;$$

In the equation, *β* is a parameter, the mean of ε is 0 and the variance obeys a normal distribution.

(2) The least square method was used to estimate the values of the parameters, and each estimated value was substituted into the linear regression model to obtain the multiple regression as shown in Equation (2):

(2) $$y=\hat{\beta_{0}}+\hat{\beta_{1}}x_{1}+\hat{\beta_{2}}x_{2}+\cdots +\hat{\beta_{n}}x_{n}\;$$

(3) According to ${\;x}_{1},x_{2},\ldots ,x_{n}$, the missing value data can be obtained.

The grey model, BP neural network model, and GM(1,1)-BP neural network model selected here are all supervised learning approaches. During the construction of these three models, there were two input variables of the model. The first variable was drug resistance data based on time series. The second variable included the type of drug used, the method of use, the duration of use, and the characteristics of the bacterial resistance mechanism. The input variable of the GM(1,1) model was the first one, and the GM(1,N) model was the second one. The neural network can apply these two methods.

With insufficient drug resistance data, only the drug resistance time series data over the years can be obtained. Therefore, k-means clustering analysis was used to mine related factors from the limited drug resistance data. This analysis considered the influence between different drugs, and took the clustering results as input variables to improve the accuracy of modeling [37].

The k-means algorithm assumes that the similarity between data is inversely proportional to the Euclidean distance between them, and clusters close data. It is assumed that each cluster can be divided into $(C_{1},C_{2},C_{3},\cdots ,C_{k}$) *k* clusters, and that the central value of each cluster can be calculated through continuous iteration to minimize the squared error *E*. The iteration is stopped when the cluster is stable [38]:

(3) $$E=\overset{K}{\underset{i=1}{\sum }}\overset{\;}{\underset{x\in C_{i}}{\sum }}{\left|\left|x-u_{i}\right|\right|}_{2}^{2}$$

In the equation, $u_{i}$ is the mean vector or the centroid of the cluster, and the expression is:

(4) $$u_{i}=\frac{1}{\left|C_{i}\right|}\overset{\;}{\underset{x\in C_{i}}{\sum }}x$$

**Figure 1:**
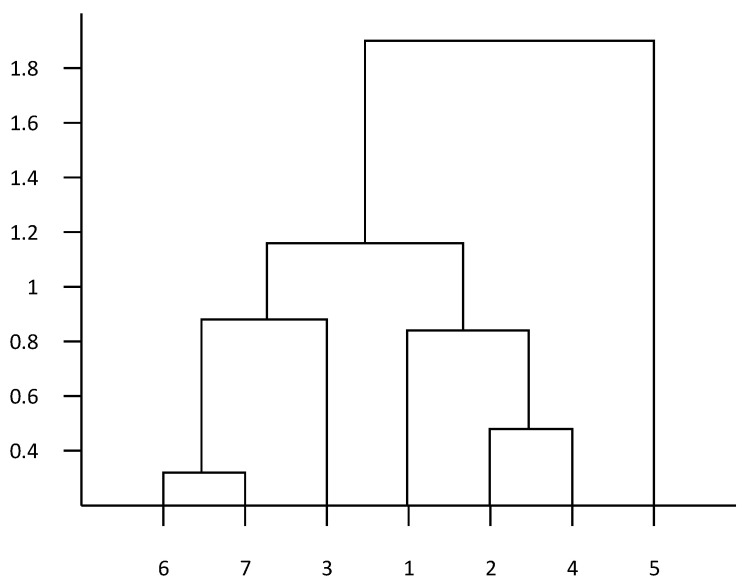
Cluster analysis of drug resistance.

As shown in Figure 1, the vertical axis indicates the correlation coefficient and the horizontal axis indicates the type of drug, 1–7 respectively, representing the seven drugs of ofloxacin, enrofloxacin, doxycycline, tetracycline, gentamicin, ceftiofur, and sulfafurazole. The drug resistance cluster analysis showed a great correlation of ceftiofur and sulfafurazole with a correlation coefficient of 0.308, and a great correlation of enrofloxacin and tetracycline with a correlation coefficient of 0.512. In the establishment of drug resistance prediction model, this clustering result can be used as input to improve the prediction accuracy of the model.

### 2.2. Methods

It is critical to select an effective and accurate drug resistance prediction method for the control of different types of drug resistance bacteria. Many mathematical models have been developed for drug resistance prediction, including exponential smoothing, grey model, Markov chain prediction, and autoregressive integral moving average models. These prediction models utilize self-historical data or similar data. This study used PyCharm, a Python integrated development environment to compare the feasibility of a grey model, a BP neural network model, and a GM(1,1)-BP neural network model for drug resistance data prediction.

#### 2.2.1. Grey Model

Grey models use the generation of discrete random numbers with obvious characteristic rules and with obviously reduced randomness to establish a model in the form of a differential equation [39,40,41]. This kind of model includes both known and unknown information, with an uncertain fuzzy relation within the system. Grey models include GM(1,1) (univariate first-order differential equation) and GM(1,N) (multi-variable first-order differential equation) [42]. During modeling, the input parameters of the model are dynamically updated to form a function that changes with time. In this way, the output error of the model is relatively small and the accuracy is relatively high in the face of time series data with more missing data. Therefore, this model is suitable for the prediction and processing of small sample data, and samples do not need to have a regular distribution.

#### 2.2.2. BP Neural Network Model

BP neural networks are multi-layer feedforward neural networks, with characteristics of input forward transmission and error backpropagation. These models are widely used for nonlinear dynamic problems such as regression prediction [43]. BP neural networks are generally composed of an input layer, a hidden layer, and an output layer. After hidden layer, layer-by-layer weighted summation, and the transformation of the transfer function, the data reaches the output layer, and the output value is obtained. The value and the actual value are then compared to calculate the error value, and this error information is back-propagated. These two processes are repeated until the error meets the expectation and the final result is output.

#### 2.2.3. GM(1,1)-BP Neural Network Model

Grey system models are suitable for single exponential growth, but these models cannot be self-fed and exhibit low prediction accuracy for short-term data with a large variation trend of drug resistance. BP neural network models can easily fall into local minima, but have the advantages of a fast learning speed, nonlinear mapping, and a high fitting accuracy. In this study, an improved model was proposed that combined these two models. An ashing layer was added in front of the neural network for data ashing treatment to weaken randomness, and an albino layer was added later for information reduction. These changes were designed to utilize the advantages of the two models and improve the accuracy of the model for the drug resistance [44,45]. The modeling process is as follows:

According to the GM(1,N) equation, the differential equation with parameters can be expressed as:

(5) $$\begin{array}{c} \frac{dx_{1}^{\left(1\right)}\left(t\right)}{dt}+b_{1}x_{1}^{\left(1\right)}\left(t\right)=b_{2}x_{2}^{\left(1\right)}\left(t\right)\\ +b_{3}x_{3}^{\left(1\right)}\left(t\right)+\cdots +b_{N}x_{N}^{\left(1\right)}\left(t\right)\\ t=1,2,\ldots n \end{array}$$

**Figure 2:**
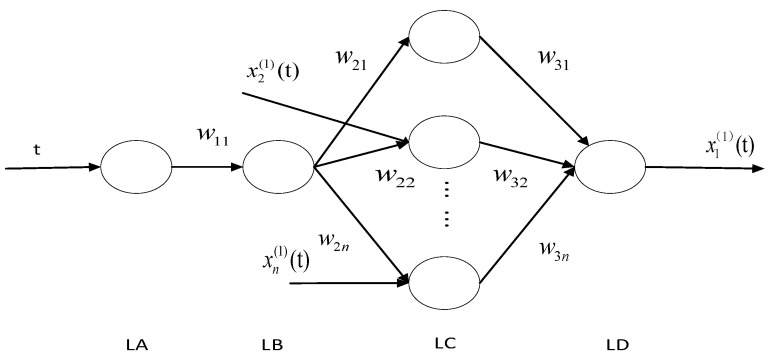
Neural network topology.

The time response equation of Equation (5) is embedded into the BP neural network with the structure shown in Figure 2, and can be written as:

(6) $$\begin{array}{c} {\hat{x}}_{1}^{\left(1\right)}\left(t\right)=\left[x_{1}^{\left(0\right)}\left(1\right)-\frac{1}{b_{1}}\overset{n}{\underset{i=2}{\sum }}b_{i}x_{i}^{\left(1\right)}\left(t\right)\right]\\ \times e^{-b\left(t-1\right)}+\frac{1}{b_{1}}\overset{n}{\underset{i=2}{\sum }}b_{i}x_{i}^{\left(1\right)}\left(t\right)\\ t=2,3,\ldots n+f \end{array}$$

In Figure 2, t is the sequence number of the input sequence,$\;x_{2}^{\left(1\right)}\left(t\right),\ldots ,x_{n}^{\left(1\right)}\left(t\right)$ is each input parameter,${\;w}_{21},w_{22},\;\ldots w_{2n},w_{31},w_{21},w_{32},\ldots w_{3n}$ are weights, LA, LB, LC, and LD represent the four-layer structure of the grey neural network and are the output values.

(1) The input parameter sequence is $b_{1},b_{2},\ldots ,b_{n}$. The initial network weight can be expressed as:

(7) $$\begin{array}{c} w_{11}=b_{1}\\ w_{21}=-x_{1}^{\left(1\right)}\\ w_{22}=\frac{2b_{2}}{b_{1}}\\ w_{23}=\frac{2b_{3}}{b_{1}}\\ w_{2n}=\frac{2b_{n}}{b_{1}}\\ w_{31}=w_{33}=\cdots =w_{3n}=1+e^{-b,t} \end{array}$$

(2) This next step is forward transfer, calculating each layer of output for each input sequence as:

LA output:

(8) $$a=w_{11}t$$

LB output:

(9) $$b_{1}=\frac{1}{e^{-w_{11}t}}$$

LC output:

(10) $$\begin{array}{c} c_{1}=bw_{21}\\ c_{2}=x_{2}^{\left(1\right)}\left(t\right)bw_{22}\\ c_{3}=x_{3}^{\left(1\right)}\left(t\right)bw_{23}\\ c_{n}=x_{n}^{\left(1\right)}\left(t\right)bw_{2n} \end{array}$$

LD output:

(11) $$d=w_{31}c_{1}+w_{32}c_{2}+\cdots +w_{3n}c_{n}-\beta x_{1}^{\left(1\right)}\left(t\right)$$

The threshold value of the LD layer output node can be expressed as:

(12) $$\beta =\left(1-e^{-b_{1}t}\right)\left(d-x_{1}^{\left(1\right)}\left(t\right)\right)$$

According to the equation:

(13) $$d=\frac{1}{b_{1}}\overset{n}{\underset{i=2}{\sum }}b_{i}x_{i}^{\left(1\right)}(t)$$

(3) Back propagation is then used to calculate the error between the output value and the expected value, and then the weight and threshold can be adjusted according to the error from LD to LB layer.

LD layer error:

(14) $$\delta =d-x_{1}^{\left(1\right)}(t)$$

LC layer error:

(15) $$\begin{array}{c} \delta_{1}=\delta \left(1+e^{-w_{11}t}\right)\\ \delta_{2}=\delta \left(1+e^{-w_{11}t}\right)\\ \ldots \\ \delta_{n}=\delta \left(1+e^{-w_{11}t}\right) \end{array}$$

LB layer error:

(16) $$\begin{array}{c} \delta_{1+n}=\frac{1}{1+e^{-w_{11}t}}\left(1-\frac{1}{1+e^{-w_{11}t}}\right)\\ \times \left(w_{21}\delta_{1}+w_{22}\delta_{2}+\cdots +w_{2n}\delta_{n}\right) \end{array}$$

The forward weight can then be adjusted according to the output value.

The LB to LC weight is changed to:

(17) $$\begin{array}{c} w_{21}=-x_{1}^{\left(1\right)}\left(0\right)\\ w_{22}=w_{22}-\frac{2b_{2}}{b_{1}}\delta_{2}b\\ \ldots \\ w_{2n}=w_{2n}-\frac{2b_{n}}{b_{1}}\delta_{n}b \end{array}$$

The LA to LB weight is changed to:

(18) $$w_{11}=w_{11}+b_{1}t\delta_{n-1}$$

The threshold is modified to:

(19) $$\begin{array}{c} \beta =\left(1+e^{-w_{11}t}\right)(\frac{w_{22}}{2}x_{2}^{\left(1\right)}(t)\\ +\frac{w_{23}}{2}x_{3}^{\left(1\right)}(t)+\cdots +\frac{w_{2n}}{2}x_{n}^{\left(1\right)}\left(t)-x_{1}^{\left(1\right)}\left(0\right)\right) \end{array}$$

(4) Next, whether or not the predicted value meets the requirements is assessed. If not, return to Step 2. If so, stop the training of the model.

## 3. Results and Discussion

### 3.1. Construction of Drug Resistance Trend Prediction Model

Based on the time series drug resistance data and the results of the cluster analysis, the grey model, BP neural network model, and GM(1,1)-BP neural network model were established to find similar drug resistance data with high correlation. These models were used to predict the resistance data, the results of the prediction were analyzed and revealed that the GM(1,1)-BP neural network model provided the most accurate prediction. Reasons for choosing the above antibiotics were that these antibiotics are widely used, and bacterial resistance to these antibiotics is high and fluctuates.

**Figure 3:**
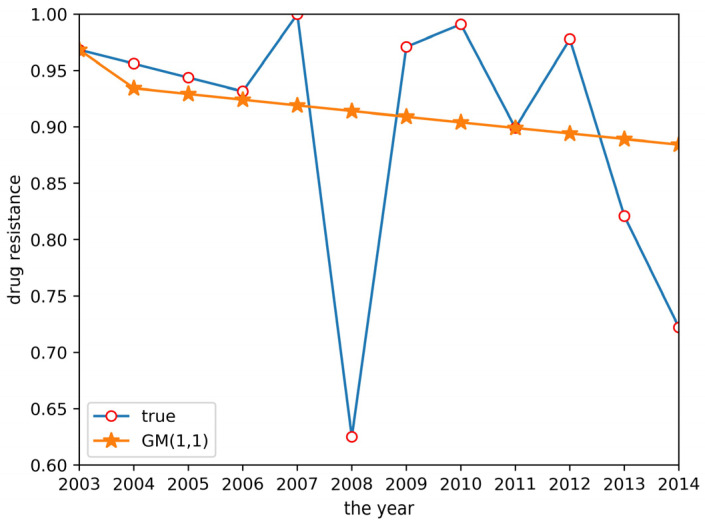
GM(1,1) prediction result.

According to the grey system theory proposed in Section 2.2.1, a GM(1,1) model was established to predict the drug resistance data for *E. coli* to sulfafurazole. The predicted results are shown in Figure 3. Although the fitting effect in the previous years was good, the prediction accuracy of the later data was low, and the prediction in 2008 and 2014 showed a big difference between the predicted and actual data. It was obvious that the GM(1,1) model did not work well enough to predict these drug resistance data.

**Figure 4:**
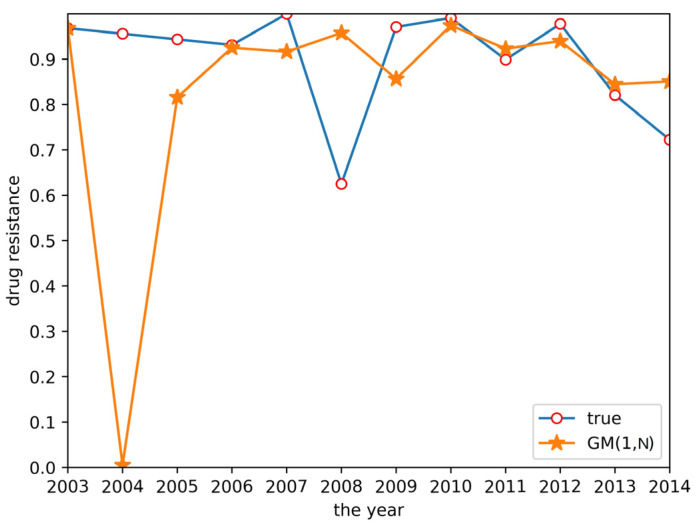
GM(1,N) prediction result.

Cluster analysis of drug resistance revealed that ceftiofur and sulfafurazole are of the same class. Both drugs have a wide range of bacterial targets and exhibit strong inhibitory effects against *E. coli*. Therefore, a GM(1,N) model was established to predict the trend of drug resistance using ceftiofur resistance rate data as the characteristic sequence and sulfafurazole as the related sequence. The results are shown in Figure 4. Relative to the large prediction error for 2004, the other years’ errors were relatively small, indicating these data can be used for short-term prediction.

**Figure 5:**
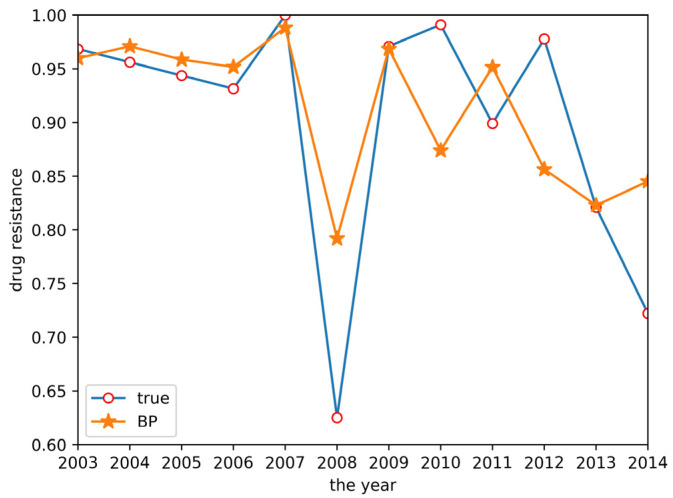
BP neural network prediction result.

As described in Section 2.2.2, a BP neural network model was established. This model used two input neurons. The hidden layer selected data for ceftiofur and doxycycline related to sulfafurazole as the input layer. The hidden layer used five input neurons, and the output layer was a single layer. Tan-sigmoid was selected as the transfer function, with a learning speed of 0.05, maximum training of 10,000 times, and a mean square error target of 0.0001. The prediction result is shown in Figure 5. The change trend of the BP neural network model is approximately the same as the true value change trend, but the prediction accuracy is low.

**Figure 6:**
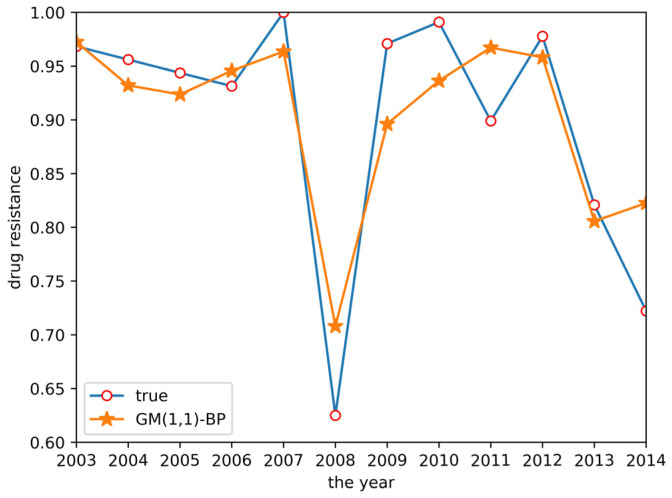
GM(1,1)-BP neural network prediction result.

Finally, a GM(1,1)-BP neural network model was established to predict drug resistance data for *E. coli* to sulfafurazole. In the established BP neural network, data for ceftiofur and doxycycline were used as input layer data, as both drugs were associated with sulfafurazole in the clustering analysis results. These data were treated by the ashing layer, and then an albino layer was added after the output results to restore the information. The prediction results are shown in Figure 6. The prediction accuracy and trend changes were similar to the true values.

### 3.2. Drug Resistance Trend Prediction Model Results

In this study, different drug resistance prediction models were used, and the predicted and experimental values for *E. coli* to sulfamethoxazole are shown in Table 1. The accuracy of the different models was compared for 2014 by comparison of the coefficient of determination (R^2^), root mean square error (RMSE), and relative error between the predicted value and true value for each of the drug resistance prediction models. The analysis results are shown in Table 2 below:

From the two tables above, GM(1,1) had a higher prediction accuracy for the trend of the initial data and GM(1,N) had a higher prediction accuracy for the trend of the later data in the time series, but the grey system models exhibited lower prediction accuracy for the overall data, with the R^2^ of both models lower than 0.5 and an RMSE higher than 0.1. The variation trend predicted by the BP neural network was similar to the true values, but with a low variation range and a low R^2^ of 0.5147, making it difficult to accurately predict the resistance data. GM(1,1) had a higher prediction accuracy for the trend of the initial data and GM(1,N) had a higher prediction accuracy for the trend of the later data in the time series, but the grey system models exhibited lower prediction accuracy for the overall data, with an R^2^ of both models lower than 0.5 and an RMSE higher than 0.1. The variation trend predicted by the BP neural network was similar to the true values, but with a low variation range, and an R^2^ of 0.5147, making it difficult to accurately predict the resistance data. Finally, the GM(1,1)-BP neural network model incorporating both the GM(1,1) and BP neural network realized an accurate estimation of drug resistance data, with an R^2^ of 0.7830 and an RMSE of only 0.0527. The relative error between the predicted result and the real value in 2014 was 13.9%. Compared with other methods, the accuracy of the GM(1,1)-BP neural network model was significantly improved, allowing for excellent drug resistance data prediction.

## 4. Design of Drug Resistance Prediction System

### 4.1. Design Goals

This study expanded on previous theoretical methods of drug resistance prediction and considered the time series characteristics of drug resistance to design a drug resistance prediction system. Historical drug resistance data were input and processed for more accurate prediction of animal drug resistance.

### 4.2. System Module

The prediction system for drug resistance of pathogenic bacteria of animal origin was built using HTML5 and Java, adopting MVC mode and using a MySQL database. 

The system function included three modules: user management, data retrieval, and drug resistance data prediction.

**Figure 7:**
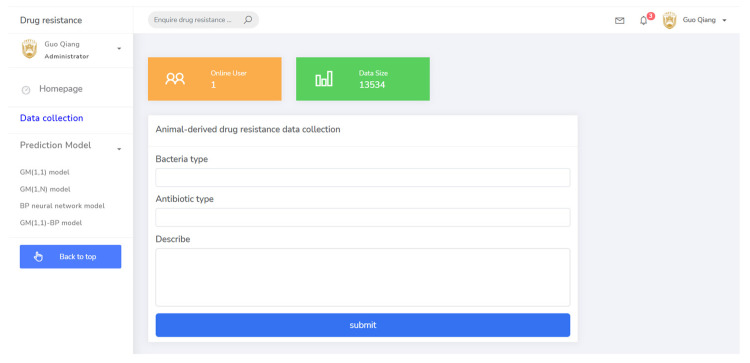
The data collection page of the system.

**Figure 8:**
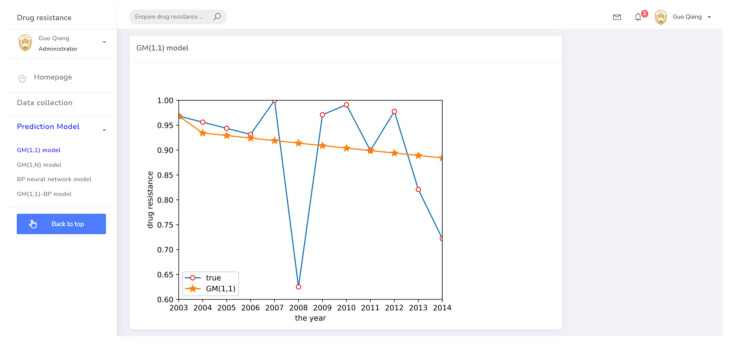
The prediction system page for the GM(1,1) model.

**Figure 9:**
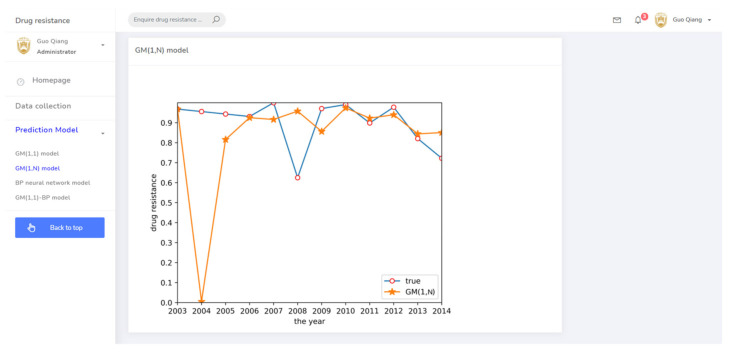
The prediction system page for the GM(1,N) model.

**Figure 10:**
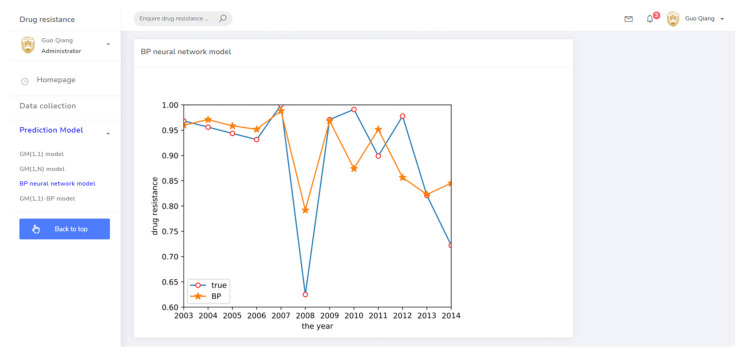
The prediction system page for the BP neural network model.

**Figure 11:**
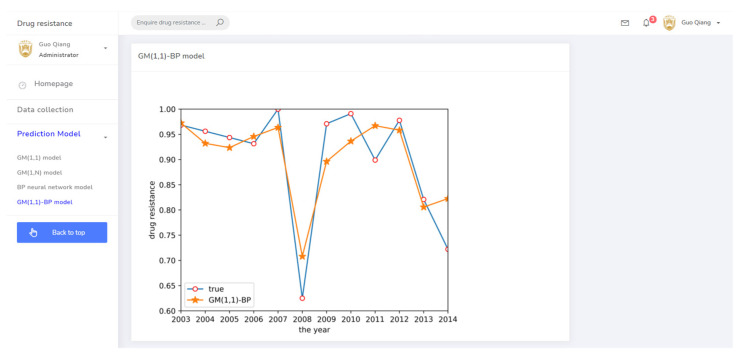
The prediction system page for the GM(1,1)-BP model.

(1) The user management module included the user login registration and the ability to receive and send information. 

(2) The basic data collection module was the basis for drug resistance monitoring, including drug information, pathogen information, time information, and other parameters. This provided basic data reference for the operation of the system and guided the steps of drug resistance prediction. The data collection page is shown in Figure 7.

(3) The drug resistance prediction module provided the trend of drug resistance of different prediction methods with time in chart form, allowing the comprehensive analysis of the drug resistance status of different strains in regional breeding animals. The related prediction pages are shown in Figure 8, Figure 9, Figure 10 and Figure 11.

## 5. Conclusions

(1) Compared with other drug resistance prediction studies, this study applied a new strategy to use data interpolation and cluster analysis to preprocess input variables, greatly improving prediction accuracy. Finding relevant similar drug resistance data, for example, using other resistance-related factor data to predict the missing data for 2014, greatly improved the prediction accuracy of the model.

(2) In this study, the classical BP neural network and the grey system model were innovatively combined to predict drug resistance data. The final relative error results were in the order of GM(1,1) > GM(1,N) > BP > GM(1,N)-BP from large to small. The relative error of the grey neural network combination model was the lowest at 13.9%. Grey model can be affected by various correlation factors. The GM(1,1)-BP neural network has better nonlinear mapping capability and high self-learning and self-adaptive ability, which will not have a great impact on the global training results after its local neurons are damaged.

(3) Based on the theoretical demonstration of the GM(1,1)-BP neural network model, a web system for drug resistance prediction was designed and developed. Using known data, this system can predict the change process of bacterial resistance to effectively decrease the spread of bacterial resistance and provide the data support to protect animal health.

## Figures and Tables

**Table 1:** Drug resistance prediction results for *E. coli* to sulfamethoxazole.

	2003	2004	2005	2006	2007	2008	2009	2010	2011	2012	2013	2014
Original value	0.9684	0.9561	0.9437	0.9314	1.000	0.6250	0.9710	0.9910	0.8990	0.9778	0.8209	0.7222
GM(1,1)	0.9684	0.9343	0.9292	0.9241	0.9190	0.9140	0.9090	0.9040	0.8990	0.8941	0.8892	0.8843
GM(1,N)	0.9684	0.0054	0.8160	0.9254	0.9164	0.9580	0.8566	0.9744	0.9231	0.9399	0.8445	0.8507
BP	0.9598	0.9710	0.9585	0.9517	0.9883	0.7920	0.9685	0.8740	0.9515	0.8564	0.8229	0.8451
GM(1,1)-BP	0.9725	0.9321	0.9236	0.9457	0.9636	0.7080	0.8962	0.9364	0.9672	0.9582	0.8057	0.8226

**Table 2:** Analysis of drug resistance prediction for the different models.

Model	R2	RMSE	Relative Error in 2014
GM(1,1)	<0.5	0.1081	0.2244
GM(1,N)	<0.5	0.2987	0.1779
BP	0.5147	0.0792	0.1701
GM(1,1)-BP	0.7830	0.0527	0.1390

## Data Availability

Data is contained within the article.
